# O-GlcNAcylation stabilizes RSK4 by antagonizing GSK3β-mediated phosphorylation to enhance radioresistance in esophageal squamous cell carcinoma

**DOI:** 10.7150/ijbs.128078

**Published:** 2026-02-04

**Authors:** Jin Liu, Duo Cao, Tianqi Xu, Junpeng Xu, Bowen Zhang, Xinya Yang, Pusong Zhao, Peng Wang, Ligang Chen, Qingge Jia, Mingyang Li

**Affiliations:** 1 Department of Pathology, Xijing Hospital and School of Basic Medicine, Fourth Military Medical University, Xi'an, China.; 2 Shaanxi Key Laboratory of Research and Utilization of Resource Plants on the Loess Plateau, College of Life Sciences, Yan'an University, Yan'an, China.; 3 Beijing National Laboratory for Condensed Matter Physics and Institute of Physics, Chinese Academy of Sciences, Beijing, China.; 4 University of Chinese Academy of Sciences, Beijing, China.; 5 Department of Clinical Medicine, School of Medicine, Yan'an University, Yan'an, China.; 6 Department of Neurosurgery, General Hospital of Northern Theater Command, Shenyang, China.; 7 Department of Reproductive Medicine, Xi'an International Medical Center Hospital, Northwest University, Xi'an, China.

**Keywords:** O-GlcNAcylation, phosphorylation, RSK4, ESCC, radioresistance

## Abstract

Esophageal squamous cell carcinoma (ESCC) is a highly lethal malignancy characterized by significant radioresistance and poor prognosis. We previously reported that ribosomal S6 protein kinase 4 (RSK4) plays a pivotal role in promoting cancer stem cell (CSC) properties and radioresistance in ESCC. This study focuses on the regulation of post-translational modifications (PTMs) of RSK4 and their effects on CSC properties and radioresistance. We demonstrate that RSK4 stability and activity are tightly regulated by phosphorylation and O-GlcNAcylation. GSK3β phosphorylates RSK4 at Thr402/Ser406, promoting its degradation via the FBXW7-dependent proteasomal pathway. Additionally, O-GlcNAcylation of RSK4 at Thr405 by OGT inhibits GSK3β-mediated phosphorylation, stabilizing RSK4 and enhancing CSC properties and radioresistance. This antagonistic relationship between phosphorylation and O-GlcNAcylation highlights a novel regulatory mechanism of RSK4 in ESCC. Moreover, targeting RSK4 O-GlcNAcylation with OSMI-4 destabilizes RSK4 and sensitizes ESCC to radiotherapy in both patient-derived xenograft and organoid models. Collectively, this study provides critical insights into the molecular mechanisms underlying ESCC radioresistance and identifies RSK4 O-GlcNAcylation as a potential therapeutic target to improve radiotherapy efficacy and overcome treatment resistance.

## Introduction

Esophageal squamous-cell carcinoma (ESCC) remains one of the most lethal malignancies worldwide, and its incidence-to-mortality ratio approaches unity^1^. China bears the greatest burden: every year the country records roughly half of all new cases and half of all ESCC-related deaths globally, and more than 95% of local esophageal cancers display squamous histology. Current imaging modalities still fail to detect early-stage disease reliably; consequently over 70% of patients present with locally advanced or metastatic tumors^2^-^4^. Although definitive chemoradiotherapy represents the cornerstone of management, intrinsic or acquired radioresistance develops in the majority of cases, and the 5-year overall survival rate after radiotherapy remains below 20%^1^, ^5^. Elucidating the molecular basis of radioresistance is therefore essential if therapeutic efficacy is to be improved.

Recent evidence implicates glycogen synthase kinase-3β (GSK3β) and ribosomal S6 kinase 4 (RSK4) in tumor initiation and progression^6^-^8^. GSK3β is a pleiotropic serine/threonine kinase that integrates multiple signaling cascades, most notably Wnt, insulin and NF-κB pathways. In the absence of Wnt ligands GSK3β constitutively phosphorylates β-catenin, thereby targeting it for proteasomal degradation; however, once the kinase is inhibited, β-catenin accumulates and drives transcription of pro-proliferative genes^9^. Additional GSK3β substrates include p53, cyclin D1 and eIF2B^10^-^12^, so its activity simultaneously modulates apoptosis, cell-cycle progression and protein synthesis. Paradoxically, GSK3β can act as either a tumor suppressor or an oncogene depending on cellular context, but its precise role in ESCC remains poorly defined.

RSK4, the least studied member of the p90 ribosomal S6 kinase family, functions as a downstream effector of Ras-MAPK signaling. The kinase participates in proliferation, differentiation and survival, and mounting data show that it is aberrantly overexpressed in several solid tumors. We previously demonstrated that RSK4 is enriched in ESCC cancer stem cells (CSC) and that high expression of RSK4 is correlates with radioresistance and poor prognosis^13^. Mechanistically, we identified RSK4-mediated phosphorylation of GSK3β at Ser9, an event that inactivates the kinase and fosters β-catenin accumulation; notably, this interaction may be reciprocal, as we also unexpectedly discovered that GSK3β can phosphorylate RSK4, suggesting the existence of a regulatory feedback loop^13^.

Post-translational modifications (PTMs) are essential biological processes that augment protein activity and expand functional diversity, where reciprocal crosstalk exists among distinct modification types. Phosphorylation and O-GlcNAcylation rank among the most vital post-translational modifications; both predominantly target serine and threonine residues of proteins, forming either competitive or synergistic regulatory relationships. Collectively, these two modifications orchestrate the precise modulation of cellular signaling cascades and serve key roles in fundamental biological processes such as stress response, metabolic homeostasis and tumorigenesis^14^-^16^. O-GlcNAcylation installs a single GlcNAc moiety onto serine or threonine residues and competes directly with phosphorylation^17^. The cycling of this sugar is catalyzed by O-GlcNAc transferase (OGT) and O-GlcNAcase (OGA), and global O-GlcNAcylation is frequently up-regulated in cancers, where it stabilizes oncoproteins, enhances invasion and promotes chemo- and radio-resistance^18^. Whether O-GlcNAcylation intersects with the RSK4-GSK3β axis in ESCC has not been explored.

The present study therefore investigates three interconnected questions: (i) how mutual phosphorylation between RSK4 and GSK3β is orchestrated; (ii) whether and where RSK4 is O-GlcNAcylated; and (iii) how these modifications collectively influence CSC traits and radioresistance. By dissecting these mechanisms, we aim to identify novel therapeutic vulnerabilities that can be exploited to sensitize ESCC to radiotherapy and ultimately improve patient survival.

## Materials and Methods

### Cell culture, reagents, and irradiation

The human ESCC cell lines TE10, TE11, ECA109, EC9706 and 293T cells were purchased from the Type Culture Collection of the Chinese Academy of Sciences (Shanghai, China). All cell lines were verified through short tandem repeat DNA profiling and were tested for mycoplasma contamination. Cells were cultured in RPMI-1640 (Gibco) or DMEM (Gibco) supplemented with 10 % FBS (Gibco) at 37 ℃ with 5 % (vol/vol) CO_2_. AR-A014418, Wortmannin, MG132 and CHX were purchased from Selleck. TMG and OSMI-4 were purchased from MedChemExpress (MCE). The cells and mice were irradiated by X-rays using the MBR-1520R-3 system (Hitachi Medico Technology, Tokyo, Japan) with the indicated dosages.

### Clinical specimens

Clinical specimens were collected from patients at Xijing Hospital. Tumor tissues and adjacent normal tissues were surgically resected and immediately snap-frozen in liquid nitrogen or fixed in 10% formalin for subsequent analysis. Patient demographics and clinical data, including age, gender, tumor stage, and treatment history, were recorded. Ethical approval was obtained from the Xijing Hospital Institutional Review Board, and informed consent was provided by all participants.

### Immunohistochemistry and evaluation

Paraffin-embedded tissues were sectioned into 4-μm slices, baked at 60 °C for 1 h, deparaffinized, rehydrated, and treated with 3% H_2_O_2_ for 10 minutes. Antigen retrieval was performed in citrate buffer at 100 °C for 15 minutes, followed by blocking endogenous peroxidase with 3% H_2_O_2_ for 15 minutes. Slides were blocked with 5% BSA for 30 minutes, incubated with antibody at 4 °C overnight, and protein conjugates were detected using the EnVision technique. Sections were counterstained with haematoxylin and visualized. RSK4 expression was scored by multiplying the percentage of positive tumor cells (1: ≤ 10%; 2: 11-50%; 3: 51-80%; 4: ≥ 81%) and staining intensity (0: none; 1: weak; 2: moderate; 3: strong). Scores ranged from 0-12, with < 7 classified as negative/low expression and ≥ 7 as positive/high expression. Two pathologists independently and blindly assessed the sections.

### Lentiviral production and transduction

To construct overexpression lentiviruses, the amplified fragments of RSK4, GSK3β, OGT, and FBXW7 were cloned into the pEZ-Lv201 vector. For shRNA construction, target sequences **(Supplementary Table 1)** were synthesized and inserted into the psi-LVRU6GP vector, with a non-targeting shRNA used as a negative control. RSK4-sgRNA lentivirus, purchased from Shanghai GeneChem, was used to knockout RSK4 in TE10 cells. ECA109 and TE10 cells were infected with concentrated viruses, and after 24 h, the supernatant was replaced with complete media, followed by puromycin selection. HA-GSK3β, Flag-RSK4, His-OGT, Myc-UBR5, Myc-FBXW7, Myc-β-TrCP, Myc-MG53, and Myc-ARIH1 plasmids were obtained from Sino Biological, while Control-Flag vector was obtained from Sigma. V5-ubiquitin and Control-V5 plasmids were obtained from our in-house bank. Three truncated variants, namely CTKD+KIM (residues 331 to 745, molecular weight 46 kDa), NTKD+CTKD (residues 1 to 683, 77 kDa), and NTKD (residues 1 to 330, 37 kDa), were constructed in the pCMV3-Flag expression vector using standard molecular biological techniques.

### Western blot

Protein samples were separated on a 10 % sodium dodecyl sulfate-polyacrylamide gel electrophoresis (SDS-PAGE) gel and then transferred to polyvinylidene difluoride (PVDF) membranes. The PVDF membranes were blocked in 5 % fat-free milk at room temperature for 1 hour. After blocking, the membranes were incubated in specific primary antibodies against different proteins at 4 °C overnight, followed by further incubation with horseradish peroxidase (HRP)-conjugated secondary antibody at room temperature for 45 minutes. Immunoreactivity was visualized by enhanced chemiluminescence (Pierce, USA) using the Molecular Imager ChemiDoc XRS^+^ System (Bio-Rad). Antibodies used in this experiment are displayed in **Supplementary Table 2**. All the western blots were quantified and the pictures are representatives of independent experiments.

### Immunofluorescence

TE10 cells were seeded and cultured in 35 mm confocal dishes (Biosharp, BS-20-GJM). After fixation with 4 % paraformaldehyde, the cells were permeabilized using 0.3 % Triton X-100 and blocked with 2 % BSA. The cells were then incubated overnight at 4 °C with primary antibodies diluted in 2 % BSA. The primary antibodies against RSK4 (Santa Cruz Biotechnology, sc-100424), FBXW7 (Santa Cruz Biotechnology, sc-293423), OGT (Proteintech, 11576-2-AP). Fluorophore-conjugated secondary antibodies (Immunoway, goat anti-mouse IgG, RS3608; Immunoway, goat anti-Rabbit IgG, RS3211), also diluted in 2 % BSA, were applied and incubated for 1 hour. Finally, the samples were mounted with Antifade Mounting Medium with DAPI (Beyotime, P0131) and imaged using a laser scanning confocal microscope.

### Caspase-3 activity assay

Caspase-3 activity was measured using a Caspase-3 Activity Assay Kit (Beyotime, C1115). Cells from each group were irradiated with 10 Gy of X-rays and routinely cultured for 24 hours. After collection, the cells were lysed with lysis buffer at a specified ratio to obtain cell lysates. The lysates were then incubated with 2 mM Ac-DEVD-pNA at 37 °C for 1 hour. The absorbance was subsequently measured at 405 nm.

### Tumor sphere-forming assay

Cells were seeded into ultra-low attachment 24-well plates at a density of 1,000 cells per well. Cells were cultured in serum-free DMEM/F12 medium (Gibco, Carlsbad, CA, USA) supplemented with 2% B27 (Invitrogen), 10 ng/mL epidermal growth factor (EGF, Invitrogen), and 10 ng/mL basic fibroblast growth factor (bFGF, Invitrogen). Subsequent sphere formation was observed. Images were captured to quantify both the diameter and number of spheres.

### Immunoprecipitation

Cell lysates were prepared in lysis buffer containing protease inhibitors. Lysates were precleared with protein A/G beads (Santa Cruz Biotechnology) for 1hour at 4 °C. The cleared lysates were then incubated with specific primary antibodies overnight at 4 °C. Subsequently, protein A/G beads were added and incubated for an additional 2 hours at 4 °C to capture the antibody-antigen complexes. The beads were subsequently washed three times with lysis buffer, and the immunoprecipitated proteins were eluted by boiling in SDS loading buffer. The eluted proteins were analyzed by western blotting.

### Clonogenic assay

Following exposure to X-ray irradiation at doses of 0, 5, or 10 Gy, cells from each group were cultured for 24 hours. The cells were then seeded into 6 cm plates and incubated for two weeks. After fixation, the cells were stained with 0.1 % crystal violet. Colony numbers were subsequently quantified and subjected to statistical analysis.

### *In vitro* glycosylation assay

RSK4 kinase, RPS6 protein, and OGT protein were purchased from Sino Biological. The 10× kinase buffer was obtained from MilliporeSigma. A 40 μL reaction mixture was prepared containing RSK4 kinase (0.2 μg), RPS6 substrate (1 μg), and OGT (1 μg). The reaction was incubated at 37 °C with gentle shaking at 220 rpm for 40 minutes. The reaction system consisted of 1× kinase buffer, 100 μmol/L ATP, 50 mM Tris-HCl (pH 7.4), 1 mM DTT, 12.5 mM MgCl_2_, 10 μM OSMI-4, and 20 μM UDP-GlcNAc. The reaction was terminated by adding 40 μL of 2× SDS loading buffer followed by boiling for 5 minutes. The phosphorylation signal of RPS6 was then detected by western blotting.

### Limiting dilution assay

Limiting dilution assays *in vivo* were performed using 4-6-week-old BALB/c nude mice, with five mice per group. Each mouse was subcutaneously injected with 1,000; 10,000; or 100,000 cells and monitored for 60 days. Tumor volume was measured every five days during this period. On day 60, the mice were euthanized, and the subcutaneous tumors were harvested. The CSC frequency was calculated using extreme limiting dilution analysis (http://bioinf.wehi.edu.au/software/elda/).

### Xenograft studies

For the xenograft tumor-formation assay, 4-6-week-old female BALB/c nude mice were used. Each mouse was subcutaneously injected with 1×10⁶ ESCC cells suspended in 200 µL of sterile PBS. For the PDX model, 4-6-week-old female non-SCID nude mice were uesd, in which with tumor fragments (2-3 mm³) from patients were implanted subcutaneously. After two generations of expansion, treatment experiments were initiated. Tumor growth was monitored twice weekly by measuring dimensions with calipers, and volume was calculated using the formula 0.5×length×width². When tumors reached 150 mm³, mice were randomized into treatment groups. OSMI-4 was administered daily at 20 mg/kg via intraperitoneal injection, while the irradiation group received 5 Gy localized X-ray irradiation on days 7 and 14 post-grouping. The BALB/c nude mouse experiment lasted 20 days, and the PDX model treatment continued for 28 days. Mice showing signs of infection or distress were promptly euthanized. At the end of the experiment, mice were euthanized, tumors were excised, photographed, weighed, and growth curves were analyzed. Tumor tissues were further processed for histopathological or molecular analysis to evaluate treatment efficacy. All procedures were performed in accordance with institutional animal care guidelines.

### ESCC organoid model

Fresh ESCC tissues were cut into small pieces and incubated in a digestion solution containing 2.5 mg/mL collagenase type Ⅱ and 10 µM Y-27632 for 1 hour, with the culture dish gently shaken every 20 minutes during incubation. The digested mixture was filtered through a 70 µm cell strainer, followed by centrifugation at 800 rpm for 5 minutes. The supernatant was discarded, and the pellet was resuspended in complete medium. Subsequently, 30 µL of the cell suspension was mixed with 30 µL of Matrigel, and the mixture was seeded into a 24-well plate at a density of 1×10⁵ cells per well, followed by incubation for 15 minutes. After the Matrigel had solidified completely, 500 µL of organoid culture medium was added to each well to establish the organoid model. On day 5, the organoids were subjected to irradiation and/or OSMI-4 treatment, and cultured for an additional 3 days to evaluate their growth status.

### Statistics analysis

All *in vitro* experiments were repeated at least 3 times. The data were expressed as mean ± SD and compared by t-test. Survival analysis was performed by Kaplan-Meier method with the log rank test using the Statistical Package for the Social Sciences (SPSS) (SPSS Inc, Chicago, IL). P values < 0.05 were considered as significant for all experiments.

## Results

### GSK3β directly phosphorylates RSK4

**Figure 1:**
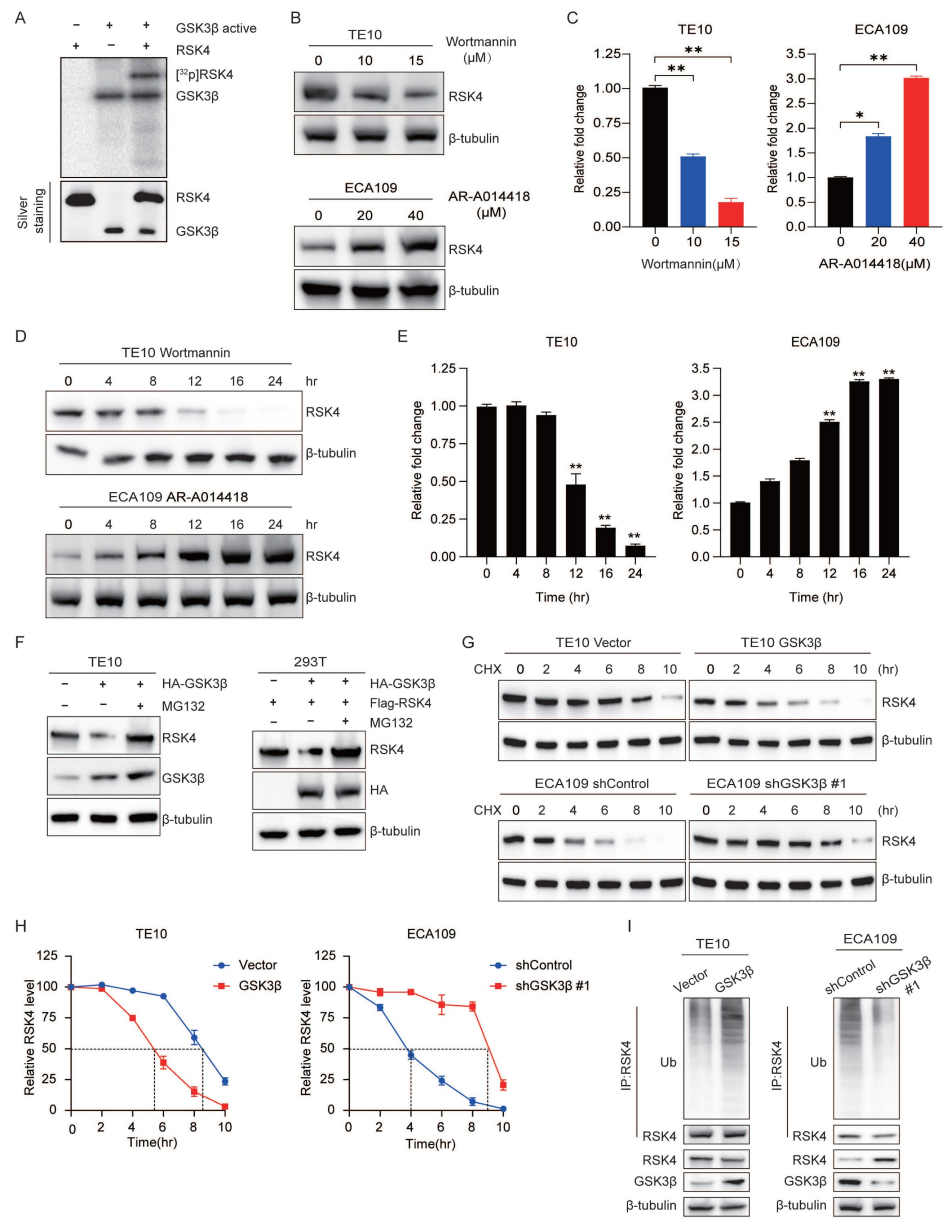
**GSK3β directly phosphorylates RSK4 in ESCC.** (A) Active GSK3β phosphorylated RSK4 *in vitro* in the presence of [γ-32^P^] ATP as visualized by an autoradiograph. The purified proteins added to each group were visualized by silver staining. (B-E) RSK4 expression was assessed by western blot in TE10 and ECA109 cells treated with either the GSK3β inhibitor AR-A014418 or the activator wortmannin at specified doses for 24 hours, or with 10 µM Wortmannin and 20 µM AR-A014418 for up to 24 hours. (F) Following GSK3β overexpression, cells were treated with MG132 to assess RSK4 protein levels. (G-H) Overexpression of GSK3β in TE10 cells or its knockdown in ECA109 cells, combined with CHX treatment, enabled comparison of RSK4 protein half-life across experimental groups. (I) Perform ubiquitination experiments in TE10 and ECA109 cells to analyze the effect of GSK3β expression on RSK4 ubiquitination levels. Data represent the mean ± SD. *P < 0.05, **P < 0.01.

While finding the downstream phosphorylation substrates of RSK4, we obtained convergent evidence that RSK4 phosphorylated GSK3β on Ser9 and thereby suppressed its kinase activity. Unexpectedly, in the course of *in vitro* kinase assays we also observed the reverse event - GSK3β phosphorylated RSK4^13^. To clarify whether this RSK4-GSK3β feedback loop operated in ESCC radio-resistance and CSC traits, we first hypothesized whether GSK3β can directly phosphorylate RSK4. Autoradiography of recombinant proteins demonstrated that GSK3β readily transferred ^32^P-γ-ATP to RSK4 *in vitro*** (Figure 1A)**.

Due to the phosphorylation of substrates by GSK3β often accompanied by proteasomal degradation^10^-^12^, accordingly, we presumed that GSK3β-mediated phosphorylation tagged RSK4 for recognition by an E3 ubiquitin ligase and subsequent proteasomal degradation. To test this model, TE10 cells were exposed to the GSK3β inhibitor AR-A014418 (AR) and ECA109 cells were expose to the GSK3β activator Wortmannin. AR increased RSK4 abundance in a concentration- and time-dependent manner, whereas Wortmannin decreased RSK4 levels under the same conditions **(Figure 1B-1E)**.

Next, GSK3β was over-expressed in TE10 cells; RSK4 protein dropped markedly, but the reduction was fully rescued by the proteasome blocker MG132. Identical results were reproduced in 293T cells with exogenous plasmids **(Figure 1F)**. Cycloheximide-chase assays revealed that GSK3β over-expression shortened the RSK4 half-life, whereas GSK3β knock-down prolonged it **(Figure 1G and 1H)**. Ubiquitination assays further showed that elevated GSK3β enhanced RSK4 poly-ubiquitination, while GSK3β silencing diminished it **(Figure 1I)**. Collectively, these data indicate that GSK3β phosphorylates RSK4, thereby governing its ubiquitin-dependent degradation and protein stability of RSK4.

### FBXW7 ubiquitinates RSK4 and triggers its degradation

**Figure 2:**
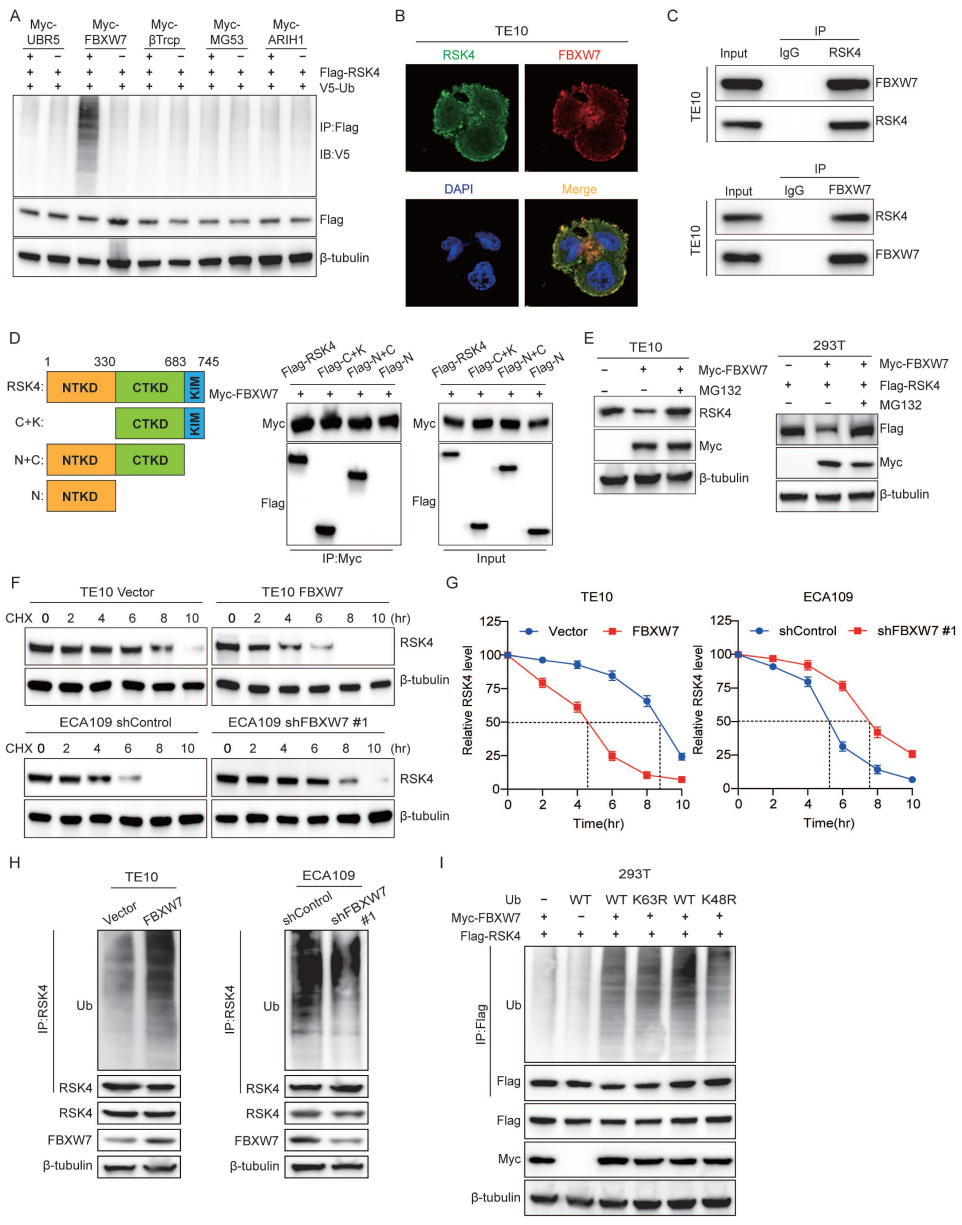
** FBXW7 is the physiological E3 ubiquitination ligase for RSK4.** (A) Transfected 293T cells with Myc-tagged UBR5, FBXW7, β-TrCP, MG53, and ARIH1 plasmids, then assessed RSK4 ubiquitination levels. (B) Colocalization of RSK4 and FBXW7 was visualized by confocal microscopy in TE10 cells. Scale bars, 100 µm. (C) The interaction of RSK4 and FBXW7 was confirmed by an endogenous co-IP assay in TE10 cells. IgG served as a negative control. (D) Mapping analyses of full-length and truncated RSK4, supported by representative co-IP assays in 293T cells, revealed that the CTKD of RSK4 mediates its interaction with FBXW7. C, CTKD; K, kinase interaction motif (KIM); N, NTKD. (E) Following FBXW7 overexpression, cells were treated with MG132 to assess RSK4 protein levels. (F-G) Overexpression of FBXW7 in TE10 cells or its knockdown in ECA109 cells, combined with CHX treatment, enabled comparison of RSK4 protein half-life across experimental groups. (H) Perform ubiquitination experiments in TE10 and ECA109 cells pre-treated with MG132 to analyze the effect of FBXW7 expression on RSK4 ubiquitination levels. (I) 293T cells transfected with Myc-FBXW7, Flag-RSK4, V5-Ub, V5-Ub K48R, and V5-Ub K63R were immunoprecipitated with Protein A/G agarose incubated with anti-Flag antibody, followed by western blotting.

To extend our observation that GSK3β governed RSK4 stability, we searched the literature for five GSK3β-interacting E3 ubiquitin ligases: UBR5, FBXW7, β-TrCP, MG53 and ARIH1^19^-^23^. We individually over-expressed each enzyme in 293T cells and performed anti-RSK4 ubiquitination assays. Only FBXW7 markedly increased the abundance of high-molecular-weight ubiquitin conjugates of RSK4 **(Figure 2A)**, implying that GSK3β might direct RSK4 toward FBXW7-mediated destruction. FBXW7 is a crucial F-box protein that belongs to the SCF-type E3 ubiquitin ligase family. Its substrates include c-MYC, NOTCH1, c-JUN, MCL-1, and FASN, all of which play pivotal roles in cellular processes such as proliferation, differentiation, apoptosis, and metabolism^24^, ^25^. As a well-characterized tumor suppressor, FBXW7 can recognize and induce the ubiquitination and degradation of these oncoproteins, thereby exerting negative regulatory effects on the signaling pathways mediated by these substrates and functioning as a key player in tumor proliferation, metastasis, and chemoresistance. To test whether FBXW7 directly regulates RSK4, we examined their physical association. Immunofluorescence staining revealed extensive co-localization of the two proteins in ESCC cells** (Figure 2B)**. Co-immunoprecipitation confirmed that endogenous FBXW7 and RSK4 interacted in these cells **(Figure 2C)**. Mapping experiments with truncated RSK4 mutants showed that FBXW7 bound specifically to the CTKD of RSK4 **(Figure 2D)**.

We next presumed whether FBXW7 controlled RSK4 abundance. Ectopic FBXW7 reduced RSK4 levels in both TE10 and 293T cells, whereas the proteasomal inhibitor MG132 restored RSK4 to baseline, indicating that FBXW7 targeted RSK4 for proteasomal degradation **(Figure 2E)**. Cycloheximide-chase assays revealed that FBXW7 over-expression shortened the half-life of RSK4, whereas FBXW7 depletion prolonged it **(Figure 2F and 2G)**. *In vivo* ubiquitination assays further demonstrated that FBXW7 enhanced RSK4 poly-ubiquitination **(Figure 2H)**.

Ubiquitin contains seven lysine residues; K48- and K63-linked chains are the most frequently studied. K48 linkages generally commit substrates to 26S proteasomal degradation, whereas K63 linkages regulate signaling and trafficking^26^, ^27^. We observed that FBXW7 exclusively assembled K48-linked ubiquitin chains on RSK4: mutation of ubiquitin at Lys48, but not at Lys63, abolished FBXW7-induced RSK4 ubiquitination **(Figure 2I)**. Thus, FBXW7 acted as the E3 ligase that installed K48-linked ubiquitin chains on RSK4 and routed the kinase for proteasomal degradation.

### GSK3β is required for FBXW7-mediated ubiquitination and degradation of RSK4

**Figure 3:**
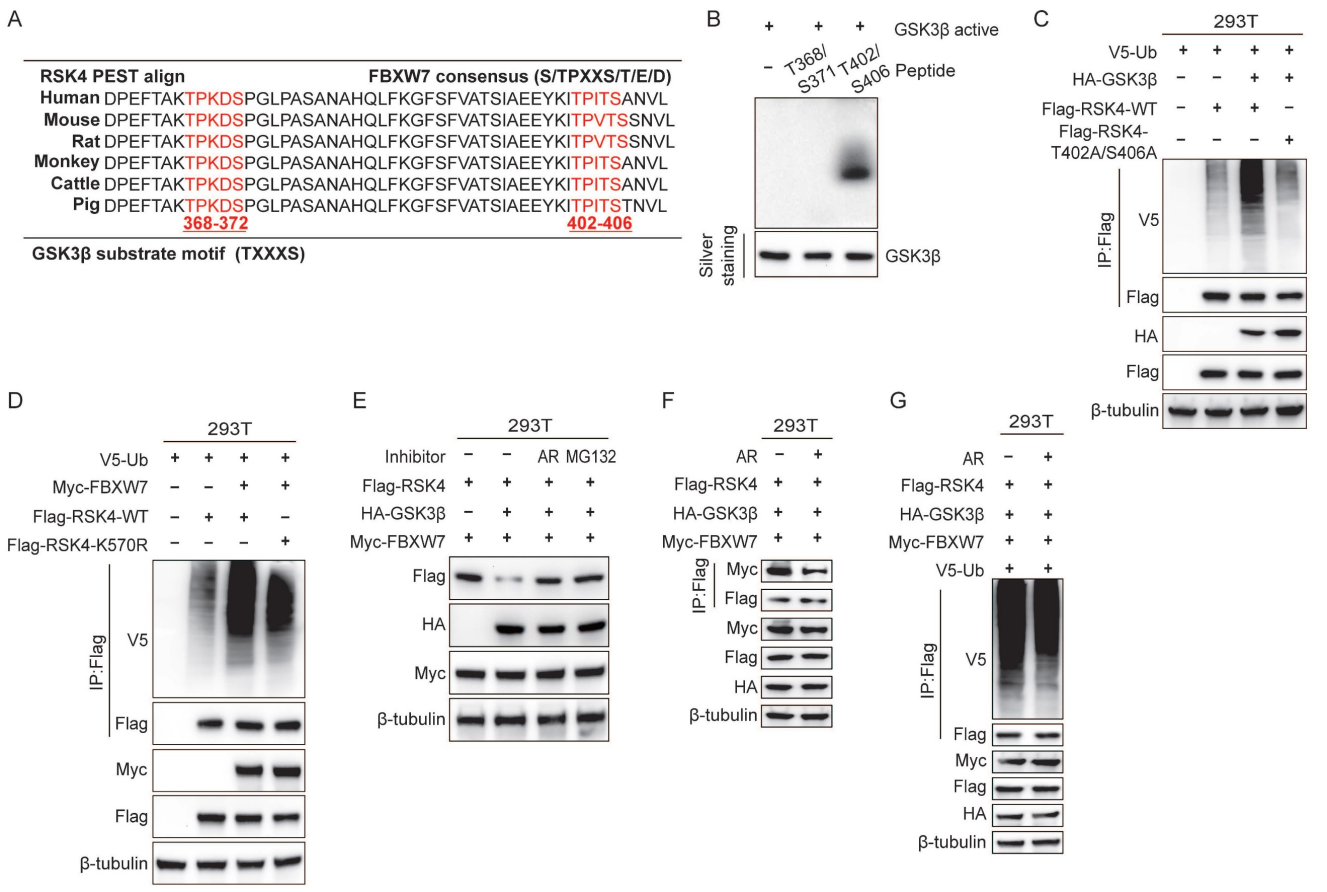
**GSK3β-mediated RSK4 phosphorylation instigates FBXW7-mediated RSK4 degradation.** (A)The RSK4 protein sequence contains two potential FBXW7 recognition sites, Thr368-Ser372 and Thr402-Ser406. The red region indicates a conserved motif within the RSK4 sequence that may be subject to FBXW7-mediated downregulation. (B) GSK3β phosphorylated RSK4 at Thr402/Ser406 *in vitro* by an autoradiograph. The input was confirmed by silver staining. (C) 293T cells were transfected with HA-GSK3β, Flag-RSK4-WT, Flag-RSK4-T402A/S406A and V5-Ub. After MG132 treatment, co-immunoprecipitation was performed using Protein A/G agarose beads incubated with anti-Flag antibody, followed by western blotting analysis with anti-V5, anti-HA and anti-Flag antibodies, respectively. (D) 293T cells were transfected with V5-Ub, Myc-FBXW7, Flag-RSK4-WT and Flag-RSK4-K570R plasmids, and cell extracts were immunoprecipitated with an anti-Flag antibody. Ubiquitinated RSK4 was detected by immunoblotting. (E-F) In 293T cells, Flag-RSK4, Myc-FBXW7 and HA-GSK3β plasmids were transfected. The cells were treated with the GSK3β inhibitor AR-A014418 and MG132 as indicated. RSK4 protein levels were detected and immunoprecipitation assays were conducted to examine the effect of GSK3β inhibition on the interaction between FBXW7. (G) 293T cells pre-treated with MG132 were transfected with the indicated plasmids, and ubiquitinated RSK4 was detected by immunoblotting.

Cross-species alignment revealed two candidate GSK3β consensus motifs (S/TXXXS/T) centered on Thr368-Ser372 and Thr402-Ser406 of RSK4 **(Figure 3A)**. An *in vitro* kinase assay showed that purified GSK3β readily phosphorylated RSK4 at Thr402 and Ser406 **(Figure 3B)**. Co-immunoprecipitation further indicated that GSK3β, enhanced RSK4 ubiquitination; notably, the T402A/S406A double mutant lost this response **(Figure 3C)**.

To investigate the effect of FBXW7 on RSK4 ubiquitination, we overexpressed FBXW7 and performed ubiquitinomic analysis in 293T cells. Mass-spectrometry-based ubiquitome profiling identified K570 as the principal ubiquitin-acceptor site on RSK4 **(Supplementary Table 3)**. Accordingly, overexpression of FBXW7 elevated the ubiquitination level of RSK4 WT, whereas RSK4 K570R attenuated RSK4 ubiquitination. These findings indicate that ubiquitination at lysine 570 (K570) is likely essential for RSK4 degradation. Notably, the RSK4 K570R failed to completely suppress RSK4 degradation, suggesting that FBXW7 may target additional ubiquitination sites on RSK4, which requires further experimental validation.

To test whether GSK3β activity linked phosphorylation to FBXW7-dependent destruction, we co-transfected Flag-RSK4, HA-GSK3β and Myc-FBXW7 into 293T cells. RSK4 levels decreased when GSK3β was present; this reduction was abolished either by the GSK3β inhibitor AR-A014418 or by MG132 **(Figure 3E)**, suggesting that phosphorylation by GSK3β would be a prerequisite for proteasomal degradation. Finally, inhibition of GSK3β diminished the FBXW7-RSK4 interaction** (Figure 3F)** and suppressed RSK4 ubiquitination** (Figure 3G)**. Collectively, these data indicated that GSK3β-phosphorylated RSK4 would create a phospho-degron that FBXW7 must recognize to trigger K48-linked ubiquitination and subsequent degradation.

### O-GlcNAcylation stabilized RSK4 and reduced its ubiquitination level

**Figure 4:**
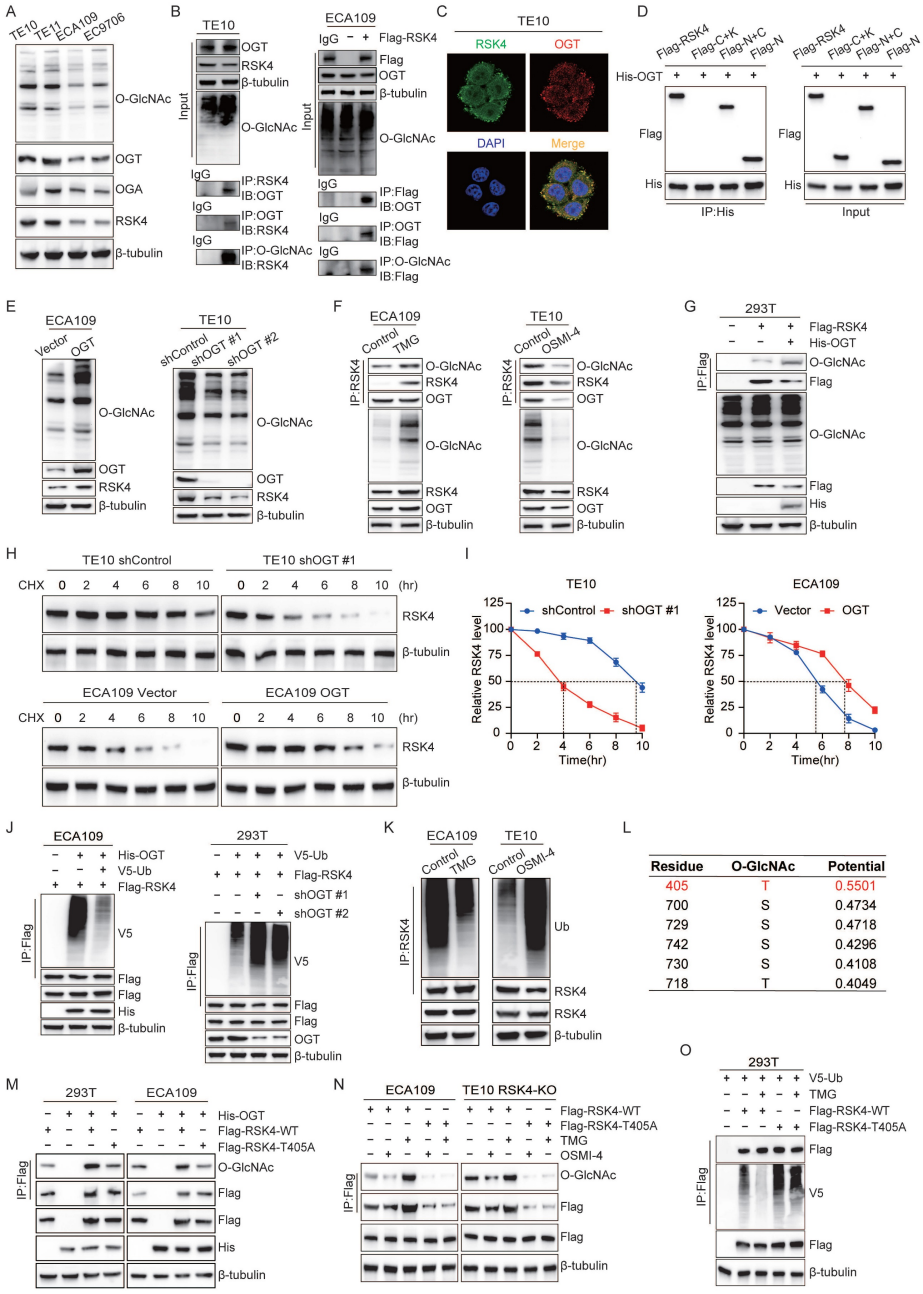
**OGT-mediated O-GlcNAcylation on Thr405 enhances RSK4 protein stability by counteracting its ubiquitination.** (A) Expression of RSK4 and O-GlcNAcylation in ESCC cell lines was detected by western blot. (B) Immunoprecipitation assays of OGT and RSK4 were examined in TE10 and RSK4-transfected ECA109 cells. (C) Subcellular colocalization of RSK4 and OGT in TE10 cells was determined by immunofluorescence staining. Scale bars, 100 µm. (D) Immunoprecipitation assays were performed in 293T cells transfected with the Flag-RSK4 and His-OGT plasmids. (E) Knockdown or overexpression of OGT regulated the protein level of RSK4 in TE10 and ECA109 cells. (F) TE10 and ECA109 cells treated with MG132, OGA inhibitor Thiamet G (TMG) and OGT inhibitor OSMI-4, and Immunoprecipitation assays were performed by anti-RSK4 antibody. (G) 293T cells were transfected with the Flag-RSK4 and His-OGT plasmids and Immunoprecipitation assays were performed by anti-Flag antibody. (H-I) Half-life and quantitative analysis of RSK4 in TE10 and ECA109 cells treated with CHX. TE10 cells stably with OGT knockdown and ECA109 cells were overexpressed OGT. (J) ECA109 and 293T cells transfected with indicated plasmids were treated with MG132, and ubiquitinated RSK4 was detected via western blot. (K) Ubiquitinated RSK4 was detected by immunoblotting in TE10 and ECA109 cells treated with MG132, OSMI-4 and TMG. (L) The YinOYang 1.2 network database predicted potential O-GlcNAcylation sites on the RSK4 protein. (M-N) 293T and ESCC cells were transfected with the indicated plasmids, and treated with MG132, TMG or OSMI-4. Immunoprecipitation assays were conducted with anti-Flag agarose beads and the indicated antibodies. (O) 293T cells were infected with V5-Ub, Flag-RSK4-WT and Flag-RSK4-T405A lentivirus and treated with TMG. Ubiquitinated RSK4 was detected by immunoblotting.

PTMs are essential regulatory mechanisms in biological systems, where phosphorylation and glycosylation are two common types that typically interact in complex ways. Just like phosphorylation, O-GlcNAcylation reversibly added a single N-acetylglucosamine moiety to serine or threonine residues, governing protein stability, subcellular trafficking and activity and thereby dictating diverse physiologic and pathologic outcomes. To explore whether O-GlcNAcylation of RSK4 contributes to its protein stability, we first surveyed four ESCC lines. O-GlcNAc global levels, as well as the transferase OGT, were higher in RSK4-high TE11 and TE10 cells than in RSK4-low ECA109 and EC9706 cells; OGA expression did not correlate with RSK4 abundance **(Figure 4A)**.

Next, endogenous and exogenous co-immunoprecipitation revealed a constitutive interaction between OGT and RSK4, and during the binding process between RSK4 and OGT, RSK4 was also O-GlcNAcylated **(Figure 4B)**. Immunofluorescence confirmed nuclear-cytoplasmic co-localization of the two proteins **(Figure 4C)**. Domain mapping showed that OGT bound selectively to the N-terminal kinase domain (NTKD) of RSK4 **(Figure 4D)**. We found that overexpression of OGT in ECA109 cells increased overall O-GlcNAcylation and upregulated RSK4 protein. Conversely, lentiviral shRNA-mediated knock-down of OGT in TE10 cells reduced global O-GlcNAcylation and concurrently lowered RSK4 protein **(Figure 4E)**. The same trend was validated when OGA inhibitors (TMG) and OGT inhibitors (OSMI-4) were used **(Figure 4F)**. After using plasmids of RSK4 and OGT in 293T cells, it was further confirmed that RSK4 can be O-GlcNAcylated **(Figure 4G)**. Cycloheximide-chase assays showed that OGT over-expression prolonged RSK4 half-life, whereas OGT depletion shortened it **(Figure 4H and 4I)**. Then, ubiquitination assays showed that elevation of O-GlcNAcylation (OGT over-expression or TMG) suppressed RSK4 poly-ubiquitination, while reduction of O-GlcNAcylation (OGT knock-down or OSMI-4) enhanced it **(Figure 4J and 4K)**. Furthermore, we investigated the effect of O-GlcNAcylation of RSK4 on its downstream substrate RPS6 by *in vitro* O-GlcNAcylation assay. The results showed that the level of p-RPS6 (S235/236) remained unchanged when OGT-mediated O-GlcNAcylation was inhibited by the addition of OSMI-4. Meanwhile, no alteration in the level of p-RPS6 (S235/236) was observed in the absence of UDP-GlcNAc, the substrate required for the O-GlcNAcylation reaction. These findings indicate that OGT-mediated O-GlcNAcylation enhances the stability of RSK4 protein but does not affect its downstream molecules **(Supplementary Figure 1)**. Taken together, these findings implied that O-GlcNAcylation can modify RSK4, thereby increasing the protein stability and reducing ubiquitination levels of RSK4.

In order to identify the specific O-GlcNAcylation modification sites of RSK4, we used the YinOYang 1.2 network database (www.cbs.dtu.dk/services/YinOYang) for prediction and identified Thr405 as the candidate with the highest score **(Figure 4L and Supplementary Table 4)**. When we substituted Thr405 with alanine (T405A), O-GlcNAcylation of RSK4 was markedly attenuated **(Figure 4M)**. Moreover, the T405A mutant no longer responded to TMG or OSMI-4: neither its O-GlcNAcylation nor its ubiquitination fluctuated upon drug treatment **(Figure 4N and 4O)**. Collectively, these findings indicated that OGT-mediated O-GlcNAcylation of Thr405 antagonized ubiquitination, thereby stabilizing RSK4 in ESCC cells.

### RSK4 O-GlcNAcylation blocks its phosphorylation by GSK3β

**Figure 5:**
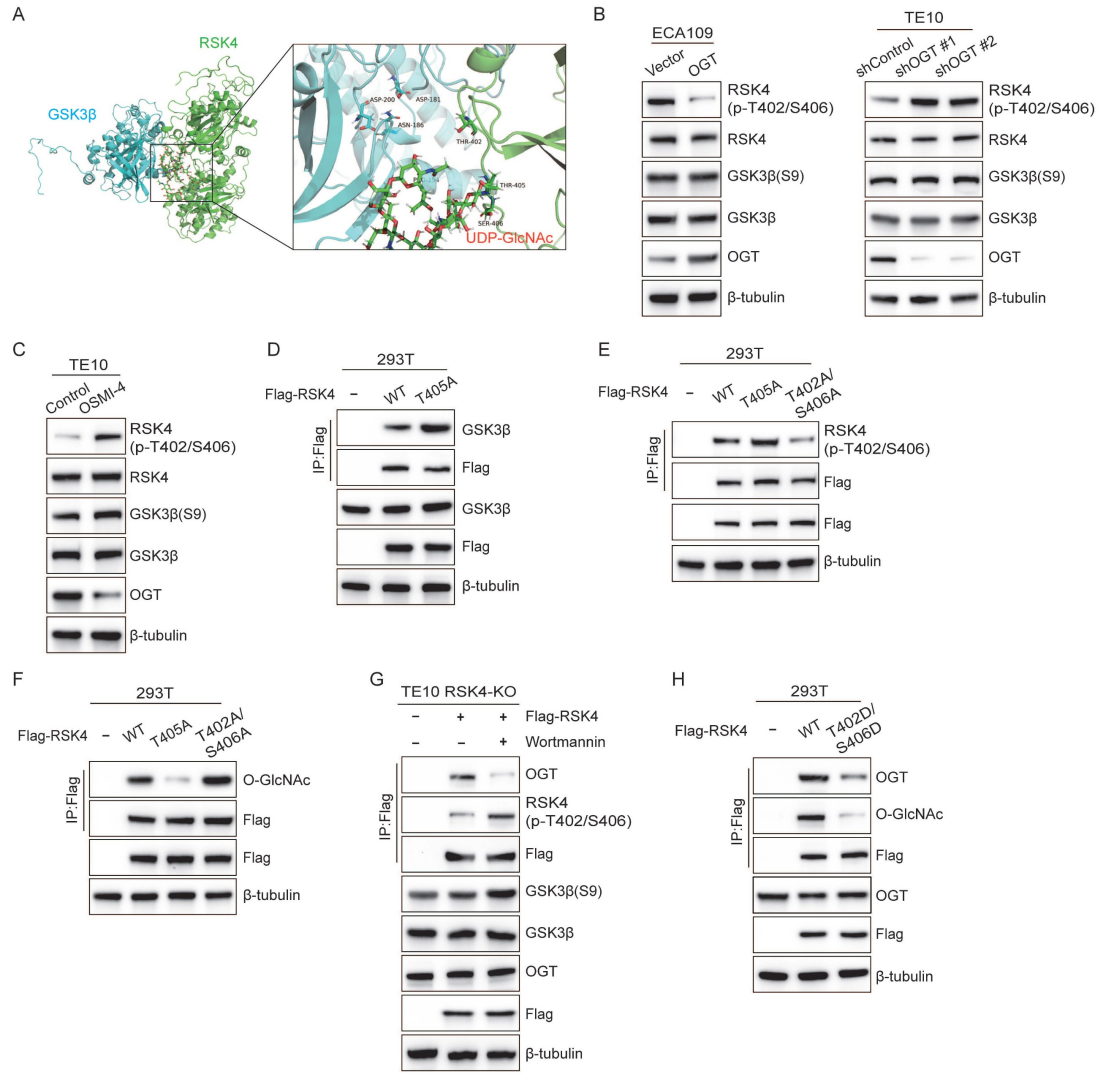
**RSK4 O-GlcNAcylation inhibits its phosphorylation by GSK3β.** (A) Structural analysis of O-GlcNAcylation-associated residues and UDP-GlcNAc binding sites in human RSK4 dimer (Uniprot ID: Q9UK32, which contains UDP-GlcNAc at both allosteric and catalytic sites). (B-C) TE10, ECA109 and 293T cells with OGT knockdown, overexpression or inhibition were treated with MG132, and analyzed with the indicated antibodies. (D) 293T cells were transfected with Flag-RSK4-WT and Flag-RSK4-T405A plasmids. Immunoprecipitation assays were carried out to examine interaction between GSK3β and RSK4. (E-F) 293T cells were transfected with Flag-RSK4-WT, Flag-RSK4-T405A and Flag-RSK4-T402A/S406A plasmids. Immunoblotting analyses were performed with anti-Flag beads to examine T402/S406 phosphorylation levels and O-GlcNAcylation of RSK4. (G) TE10 cells knockout of RSK4 were infected with Flag-RSK4 lentivirus and treated with wortmannin. Immunoprecipitation assays examined OGT and T402/S406 phosphorylation levels of RSK4. (H) Flag-RSK4-WT, Flag-RSK4-T405A and Flag-RSK4-T402D/S406D plasmids were transfected into 293T cells. Immunoblotting analyses were conducted with indicated antibodies by Immunoblot analyses.

To clarify the interaction between O-GlcNAcylation site at Thr405 and phosphorylation site at Thr402/Ser406, we performed 50ns molecular-dynamics refinement. Although the O-GlcNAc moiety did not fully occlude the GSK3β catalytic cleft, the O-GlcNAc moiety and the phosphate sites lay only a few angstroms apart, suggesting that O-GlcNAcylation could sterically hinder subsequent phosphorylation **(Figure 5A)**. Encouraged by these in-silico predictions, we next asked whether the modifications compete inside cells.

To track the phospho-signal, we raised a phospho-specific antibody against p-RSK4 (Thr402/Ser406) **(Supplementary Figure 2)**. Strikingly, when OGT was either silenced or inhibited with OSMI-4, level of p-RSK4 (Thr402/Ser406) increased markedly. Conversely, OGT over-expression suppressed the same phospho-epitope without altering total GSK3β or its inhibitory Ser9 phosphorylation **(Figure 5B and 5C)**, implying that O-GlcNAcylation of RSK4 actively antagonizes phosphorylation rather than modulating the kinase itself.

Then we dissected the interplay genetically. Compared with the wild-type RSK4, the O-GlcNAcylation-defective T405A mutant bound GSK3β more avidly and displayed robustly elevated p-RSK4 (Thr402/Ser406) **(Figure 5D and 5E)**, whereas the phosphorylation-defective T402A/S406A mutant became heavily O-GlcNAcylated **(Figure 5F)**. Thus, blocking one mark reciprocally enriched the other, suggesting the mutually exclusive relationship between phosphorylation and O-GlcNAcylation of RSK4. In addition, we activated GSK3β with Wortmannin, which resulted in upregulation of p-RSK4 (Thr402/Ser406) levels and significantly reduced the binding of RSK4 to OGT** (Figure 5G)**. Finally, the phospho-mimetic T402D/S406D form weakened the interaction with OGT and resisted O-GlcNAcylation of RSK4 **(Figure 5H)**. Taken together, these data argue that RSK4 Thr405 O-GlcNAcylation and Thr402/Ser406 phosphorylation constitute a mutually exclusive binary switch.

### RSK4 O-GlcNAcylation and phosphorylation regulate the CSC properties and radioresistance in ESCC

To evaluate whether targeting RSK4 O-GlcNAcylation and phosphorylation affects ESCC behavior, we first generated TE10 cells in which the endogenous RSK4 allele had been disrupted by CRISPR/Cas9. Subsequently, these knockout cells were reconstituted with lentiviruses encoding RSK4-WT, RSK4-T405A, RSK4-T402A/S406A, or RSK4-T402D/S406D.

**Figure 6:**
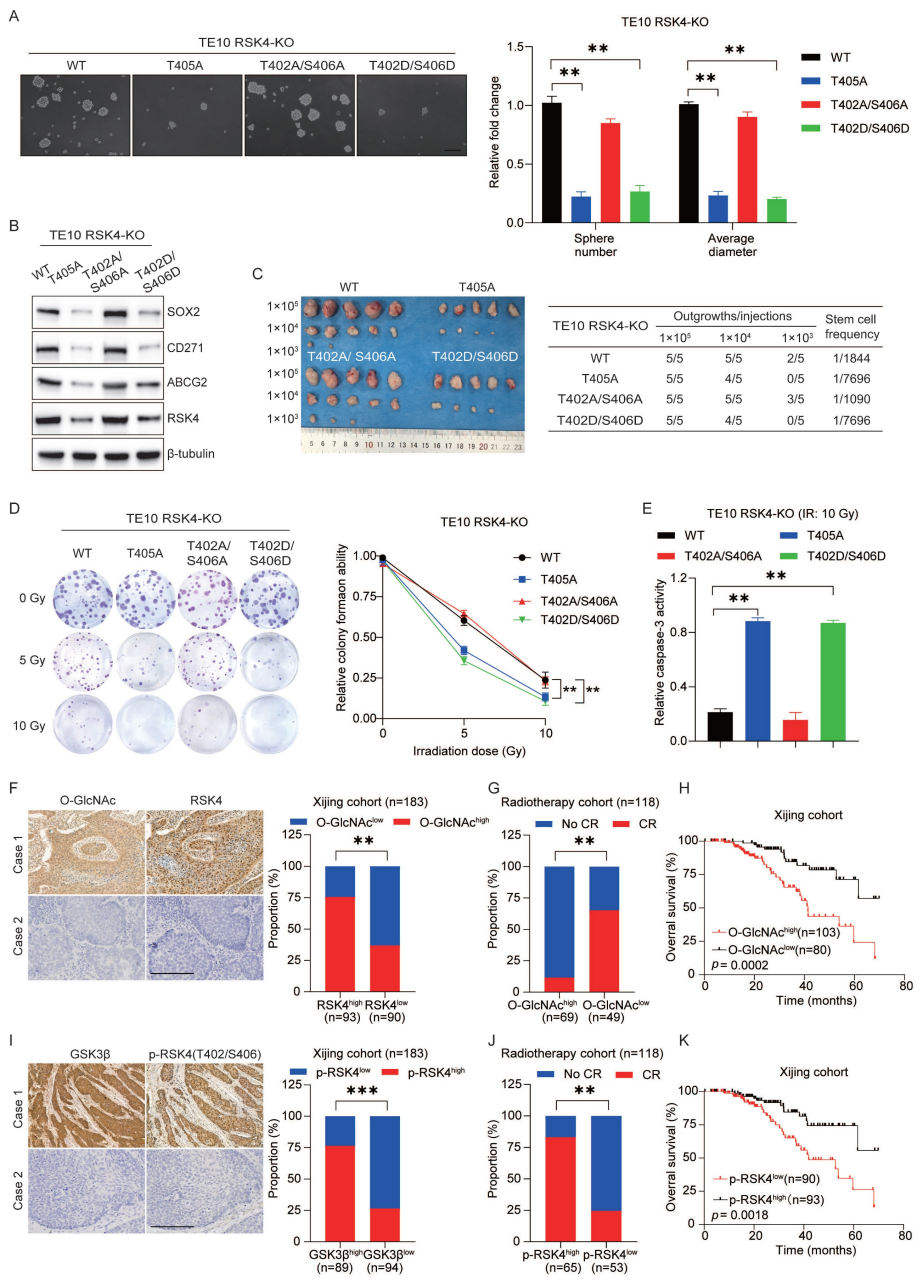
**RSK4 O-GlcNAcylation and phosphorylation regulate the CSC properties and radioresistance in ESCC.** (A) TE10 cells knockout of RSK4 were introduced with Flag-RSK4-WT, Flag-RSK4-T405A, Flag-RSK4-T402DA/S406A and Flag-RSK4-T402D/S406D lentivirus. Sphere formation assay showed the sphere-forming ability of each group. Scale bars, 100 µm. (B) TE10 cells knockout of RSK4 were introduced with the indicated lentivirus and western blot performed to verify ESCC CSC markers. (C) Limiting dilution analysis showed cells with expression of RSK4-WT and RSK4-T402A/S406A had higher tumorigenicity compared with RSK4-T405A and RSK4-T402D/S406D group. (D-E) Clonogenic survival assays and Relative caspase-3 activity of TE10 cells with expression of RSK4-WT, RSK4-T405A, RSK4-T402A/S406A and RSK4-T402D/S406D at the indicated irradiation doses. (F) Representative IHC images of O-GlcNAcylation and RSK4 protein expression in patients with ESCC. Scale bar: 100 μm. Histograms show the correlation of the IHC data for high or low RSK4 expression relative to the level of O-GlcNAcylation. (G) The higher level of O-GlcNAcylation, the lower the complete response rate to radiotherapy. (H) Kaplan-Meier estimation of the overral survival for 183 patients with ESCC treated with radiotherapy according to O-GlcNAcylation levels in the primary tumor. (I) IHC images depict GSK3β and T402/S406 phosphorylation levels of RSK4 in ESCC patient samples (scale bar: 100 μm). Histograms illustrate the correlation between IHC results for high or low GSK3β expression and corresponding T402/S406 phosphorylation level of RSK4. (J) The lower T402/S406 phosphorylation levels of RSK4 was, the lower the complete response rate to radiotherapy. (K) Kaplan-Meier analysis estimates overall survival for 183 ESCC patients receiving radiotherapy, stratified by T402/S406 phosphorylation level of RSK4 in the primary tumor. Data represent the mean ± SD. **P < 0.01, ***P < 0.001.

Compared with the wild-type RSK4, impairment of O-GlcNAcylation (T405A) or elevation of phosphorylation (T402D/S406D) markedly reduced tumor spheroid formation and expression levels of CSC markers SOX2, CD271 and ABCG2, whereas blockade of phosphorylation (T402A/S406A) up-regulated these markers and augmented tumor-sphere formation **(Figure 6A and 6B)**. In addition, limited dilution transplantation experiments confirmed that the ability to initiate tumors *in vivo* decreases when RSK4 O-GlcNAcylation is insufficient or when phosphorylation is enhanced **(Figure 6C)**. Moreover, radioclonal survival assays revealed that the absence of O-GlcNAcylation (T405A) or forced phosphorylation (T402D/S406D) reduced the number of colonies, while the prevention of phosphorylation (T402A/S406A) increased the number of colonies** (Figure 6D)**. Similarly, caspase-3 activity measurements also showed the same trend: cells carrying T405A or S402D/T406D showed increased caspase-3 cleavage and apoptosis, while the S402A/T406A mutant resisted apoptosis **(Figure 6E)**.

For further investigation, biopsy samples from ESCC patients were collected for IHC staining analysis of RSK4 and O-GlcNAcylation expression. Our results revealed that samples with high RSK4 expression exhibited correspondingly high levels of O-GlcNAcylation, whereas specimens with low RSK4 expression displayed reduced O-GlcNAcylation levels** (Figure 6F)**. Moreover, we collected biopsy samples from ESCC patients who underwent radical radiotherapy and revealed that ESCC patients with high O-GlcNAc levels exhibited stronger resistance to radiotherapy and lower survival rate compared to those with low expression **(Figure 6G and 6H)**. Analysis of the relationship between the level of O-GlcNAc and various clinical pathological indicators revealed that its level positively correlates with lymph node metastasis and vascular invasion **(Supplementary Table 5)**. Multivariate regression survival analysis identified the level of O-GlcNAc as an independent prognostic factor for ESCC **(Supplementary Table 6)**. Also, we found that the proportion of samples with high p-RSK4 (Thr402/Ser406) levels was significantly increased in specimens with high GSK3β expression, whereas the opposite trend was observed in samples with low GSK3β expression **(Figure 6I)**. ESCC patients with high p-RSK4 (Thr402/Ser406) levels showed weaker resistance to radiotherapy and higher survival rate compared to those with low expression **(Figure 6J and 6K)**. Furthermore, the level of p-RSK4 (Thr402/Ser406) was found to negatively correlate with lymph node metastasis and vascular invasion **(Supplementary Table 7)**. Multivariate regression survival analysis identified p-RSK4 (Thr402/Ser406) also constitutes an independent prognostic factor for ESCC **(Supplementary Table 6)**. Collectively, these data indicate that O-GlcNAcylation and phosphorylation of RSK4 can mutually exclusivey regulate the CSC properties and radioresistance of ESCC cells.

### Targeting OGT-mediated RSK4 O-GlcNAcylation improves the radiosensitivity of ESCC

**Figure 7:**
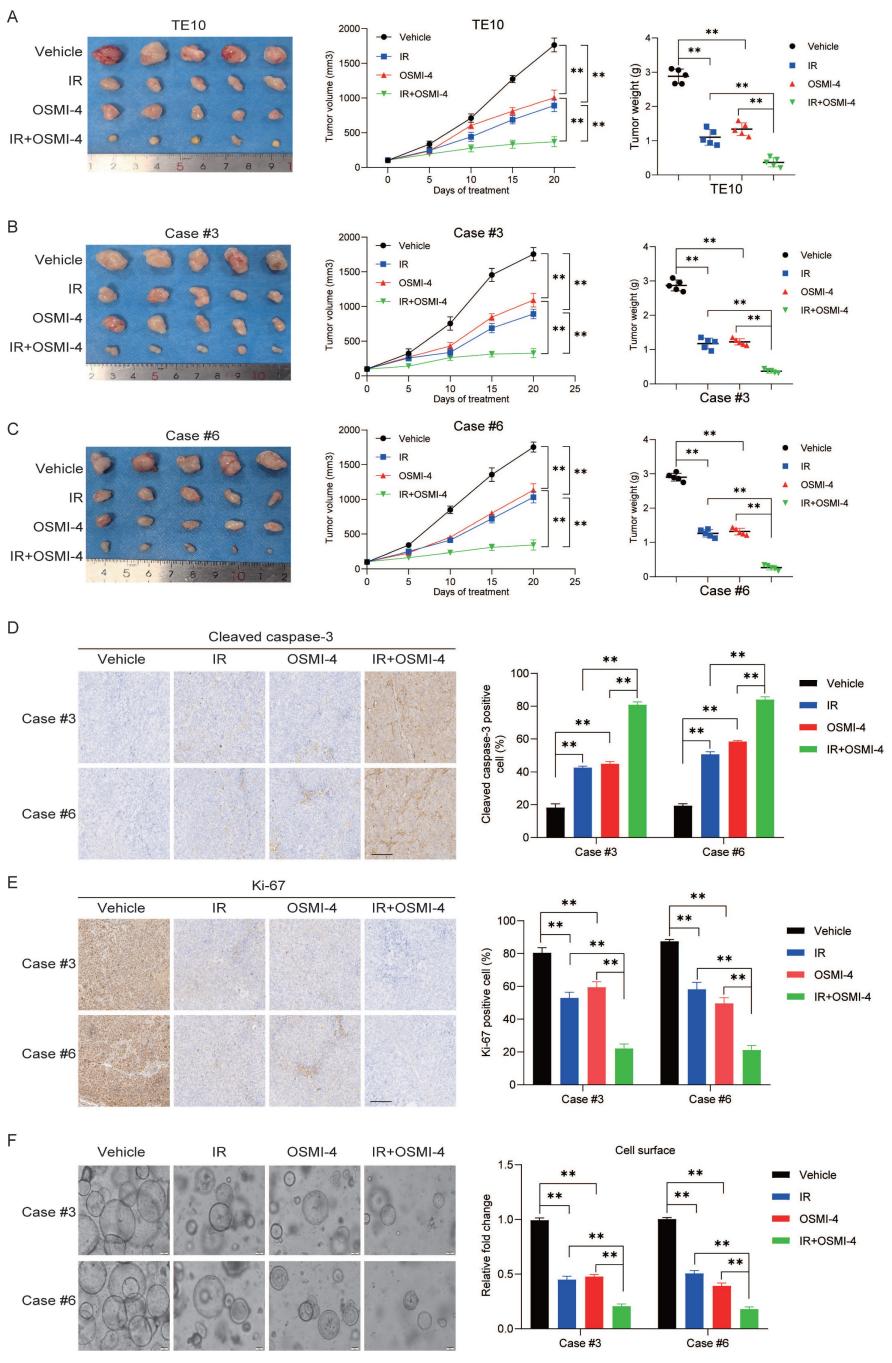
**Targeting OGT-mediated RSK4 O-GlcNAcylation improves the radiosensitivity of ESCC.** (A) TE10 cells were injected into nude mice and treated with vehicle, OSMI-4 (20 mg/kg/day, i.p. injection), and/or IR (5 Gy, twice) (n=5 per group). Representative images of the resulting subcutaneous tumors are shown. Tumor growth curves and final average tumor weights are presented. (B-C) PDX tumors case #3 and #6 treated with the vehicle, OSMI-4 (20 mg/kg/day, i.p. injection), and/or IR (5 Gy, twice) (n=5 per group). The growth curve of the tumor size and average tumor weight are presented. (D-E) IHC analyses of cleaved caspase-3 and Ki-67 in the indicated groups. Scale bars: 100 μm. (F) Representative images and the calculated surface area of ESCC organoids treated with the vehicle, OSMI-4 (10 μM), and/or IR (5 Gy) are presented. Data represent the mean ± SD. **P < 0.01.

To further explore the role of targeted inhibition of RSK4 O-GlcNAcylation in radiotherapy for ESCC, we established a subcutaneous xenograft tumor model in nude mice via subcutaneous inoculation of TE10 cells. Combined treatment with irradiation and OSMI-4 showed a stronger suppressive effect on TE10 cell proliferation than irradiation or OSMI-4 alone, leading to a significant reduction in tumor growth **(Figure 7A)**. Moreover, two high-RSK4-expressing ESCC PDX models (Case #3 and Case #6) were chosen from our previously established valid ESCC PDX models for combined treatment with OSMI-4 and irradiation to assess therapeutic responses 13. The results showed that OSMI-4 plus irradiation induced a more potent suppression of ESCC growth than either treatment alone, with significantly decreased Ki-67 proliferation index and increased cleaved caspase-3 expression **(Figure 7B-7E)**. Concordantly, identical results were observed when tumor tissues were cultured as organoids and treated with irradiation and OSMI-4 **(Figure 7F)**. Collectively, these findings indicate that OSMI-4 can enhance the radiosensitivity of ESCC cells, and the combination of OSMI-4 and irradiation exerts a synergistic effect to improve the therapeutic efficacy against ESCC tumors.

## Discussion

ESCC continues to pose a significant therapeutic challenge due to its marked radioresistance and unfavorable prognosis^28^, ^29^. In this study, we elucidate the pivotal role of RSK4 in driving CSC properties and radiotherapy resistance through the dynamic interplay of PTMs. Our findings demonstrate that RSK4 stability and activity are tightly regulated by phosphorylation and O-GlcNAcylation, which inversely modulate its degradation and oncogenic functions. The identification of RSK4 as a convergence point for these PTMs provides critical insights into the molecular mechanisms underlying ESCC radioresistance and highlights potential therapeutic vulnerabilities.

Our previous study demonstrated that RSK4 can directly phosphorylate GSK3β at Ser9, thereby inhibiting its activity and promoting β-catenin nuclear translocation to activate the Wnt signaling pathway^13^. This process enhances both the tumorigenicity and radioresistance of ESCC. Intriguingly, our *in vitro* kinase assays further revealed that GSK3β can reciprocally phosphorylate RSK4. Many oncoproteins, such as β-catenin, Snail, and Mcl-1, are phosphorylated by GSK3β and subsequently targeted for proteasomal degradation by E3 ubiquitin ligases^30^-^34^. In this study, we confirmed that GSK3β can directly phosphorylate RSK4 at Thr402/Ser406, thereby affecting its protein stability. Thus, in ESCC, a double negative feedback loop exists between RSK4 and GSK3β: RSK4 phosphorylates GSK3β at Ser9, inhibiting GSK3β activity, while GSK3β phosphorylates RSK4 at Thr402/Ser406, promoting its degradation via the proteasomal pathway. Conversely, when GSK3β is phosphorylated and inactivated by RSK4, RSK4 stability is increased. Clinical ESCC samples exhibited a positive correlation between p-RSK4 (Thr402/Ser406) levels and GSK3β levels. We further identified FBXW7 as the E3 ubiquitin ligase responsible for RSK4 degradation. After GSK3β phosphorylates RSK4, FBXW7 induces its ubiquitination and subsequent proteasomal degradation.

Another key finding of this study is the antagonistic relationship between GSK3β-mediated phosphorylation and OGT-mediated O-GlcNAcylation of RSK4. PTMs are essential regulatory mechanisms in biological systems, encompassing a wide variety of types^35^-^37^. Among these, phosphorylation and O-GlcNAcylation are two common types that often interact in complex ways. Specifically, GSK3β phosphorylates RSK4 at Thr402/Ser406, creating a phospho-degron that is recognized by the E3 ubiquitin ligase FBXW7. This leads to RSK4 ubiquitination and subsequent proteasomal degradation. Conversely, O-GlcNAcylation at Thr405 stabilizes RSK4 by sterically hindering GSK3β-mediated phosphorylation and the subsequent FBXW7-dependent degradation. This competitive modification at adjacent residues (Thr405 versus Thr402/Ser406) highlights a novel regulatory mechanism where metabolic signaling (via O-GlcNAcylation) counteracts kinase-driven proteolysis. This interplay is consistent with growing evidence that PTMs like phosphorylation and O-GlcNAcylation often compete for shared Ser/Thr residues^38^, dynamically modulating protein stability and function in response to cellular stress.

O-GlcNAcylation primarily takes place in the cytoplasm and nucleus, rather than in the endoplasmic reticulum or Golgi apparatus^39^, ^40^. Functionally, O-GlcNAcylation can compete with phosphorylation for the same protein site, stabilize folded protein structures, promote glycoprotein folding, and regulate signaling pathways such as NF-κB and p53^39^, ^41^, ^42^. Numerous studies have shown that O-GlcNAcylation levels are elevated in various tumors, and this increase is closely associated with tumor cell growth, survival, metabolism, and treatment resistance^17^, ^43^-^47^. Many studies have linked O-GlcNAcylation to the development of treatment resistance in tumors^48^-^50^.

In this study, we found that OGT-mediated O-GlcNAcylation of RSK4 at Thr405 inhibited GSK3β-induced phosphorylation of RSK4, thereby enhancing RSK4 protein stability and ultimately enhancing CSC properties and radioresistance in ESCC. By stabilizing RSK4, O-GlcNAcylation amplifies its oncogenic role in ESCC, indicating that RSK4 O-GlcNAcylation promotes the expression of CSC markers, spheroid formation, and *in vivo* tumorigenicity, while impairing apoptosis post-irradiation. These effects are reversed by RSK4 phosphorylation, which accelerates its degradation and weakens its tumorigenic effect. These findings suggest that targeting RSK4 O-GlcNAcylation holds promise as a novel strategy to enhance ESCC radiosensitivity and overcome radioresistance. Preclinical models demonstrate that inhibiting OGT with OSMI-4^51^-^53^ destabilizes RSK4, sensitizing ESCC to radiotherapy. The synergistic effect of OSMI-4 and irradiation on reducing tumor growth and enhancing apoptosis underscores the therapeutic potential of combining OGT inhibitors with conventional radiotherapy. Furthermore, the identification of Thr405 as the primary O-GlcNAcylation site offers a rationale for developing site-specific inhibitors to disrupt RSK4 stability without globally altering O-GlcNAc dynamics. Our previous *in vivo* studies confirmed that BI-D1870, a specific RSK4 inhibitor, could enhance the radiosensitivity of ESCC cells and exert remarkable tumor-inhibitory effects on ESCC PDX models. In this study, we further found that the combination of OSMI-4 and radiotherapy exerts robust therapeutic efficacy in both ESCC cells and ESCC PDX models. Nevertheless, specific inhibitors targeting either RSK4 O-GlcNAcylation or the Thr405 O-GlcNAcylation site of RSK4 remain unavailable to date. Considering the pivotal role of RSK4 in driving the malignant progression of ESCC, the development of highly efficient and selective O-GlcNAcylation inhibitors against RSK4 is urgently required for future ESCC targeted therapy.

In summary, this study focuses on investigating the role and mechanism of O-GlcNAcylation and phosphorylation modifications of RSK4 in ESCC. We have demonstrated that O-GlcNAcylation of RSK4 inhibits GSK3β-mediated phosphorylation and ubiquitin-dependent degradation, enhances protein stability, and contributes to CSC properties and radioresistance in ESCC cells. This study holds significant clinical implications for addressing the challenges of treatment resistance and recurrence in ESCC, and provides a new theoretical basis and potential molecular targets to improve radiotherapy efficacy and promoting the development of precision and personalized therapies for ESCC. Of course, these findings still need to be further verified and expanded in larger clinical samples and more in-depth mechanistic studies to achieve eventual clinical application translation.

## Supplementary Material

Supplementary figures and tables 1,2, 4-7.

Supplementary table 3.

## Data Availability

Please contact the corresponding author for data requests.
