# Supplementary material for: O-GlcNAcylation stabilizes RSK4 by antagonizing GSK3β-mediated phosphorylation to enhance radioresistance in esophageal squamous cell carcinoma
Source: Int J Biol Sci. 2026 Feb 4;22(5):2418–34. doi: 10.7150/ijbs.128078 (PMC12965142; doi:10.7150/ijbs.128078)
Supplement: Supplementary file 1 — Supplementary figures and tables 1,2, 4-7. [file ijbsv22p2418s1.pdf]

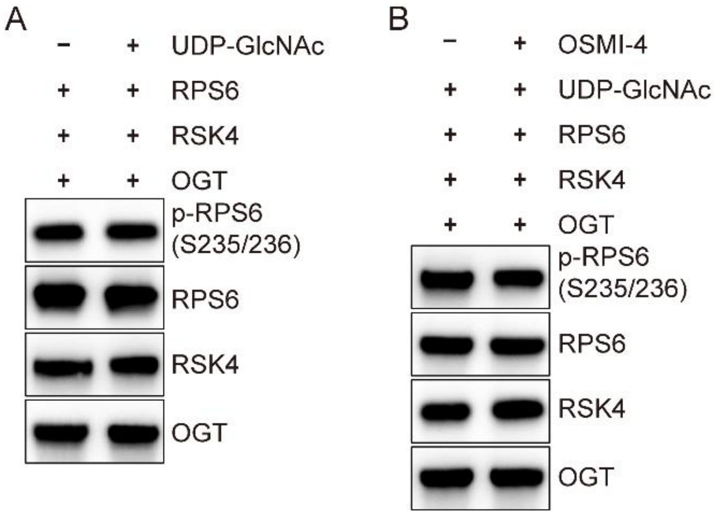

Supplementary Figure 1. O-GlcNAcylation of RSK4 had no effect on the expression of its downstream substrates. (A) In *in vitro* glycosylation assay, the level of p-RPS6 (S235/236) remained unchanged in the absence of the O-GlcNAcylation substrate UDP-GlcNAc. (B) *In vitro* glycosylation assay results showed that the level of p-RPS6 (S235/236) remained unchanged when the OGT inhibitor OSMI-4 was added.

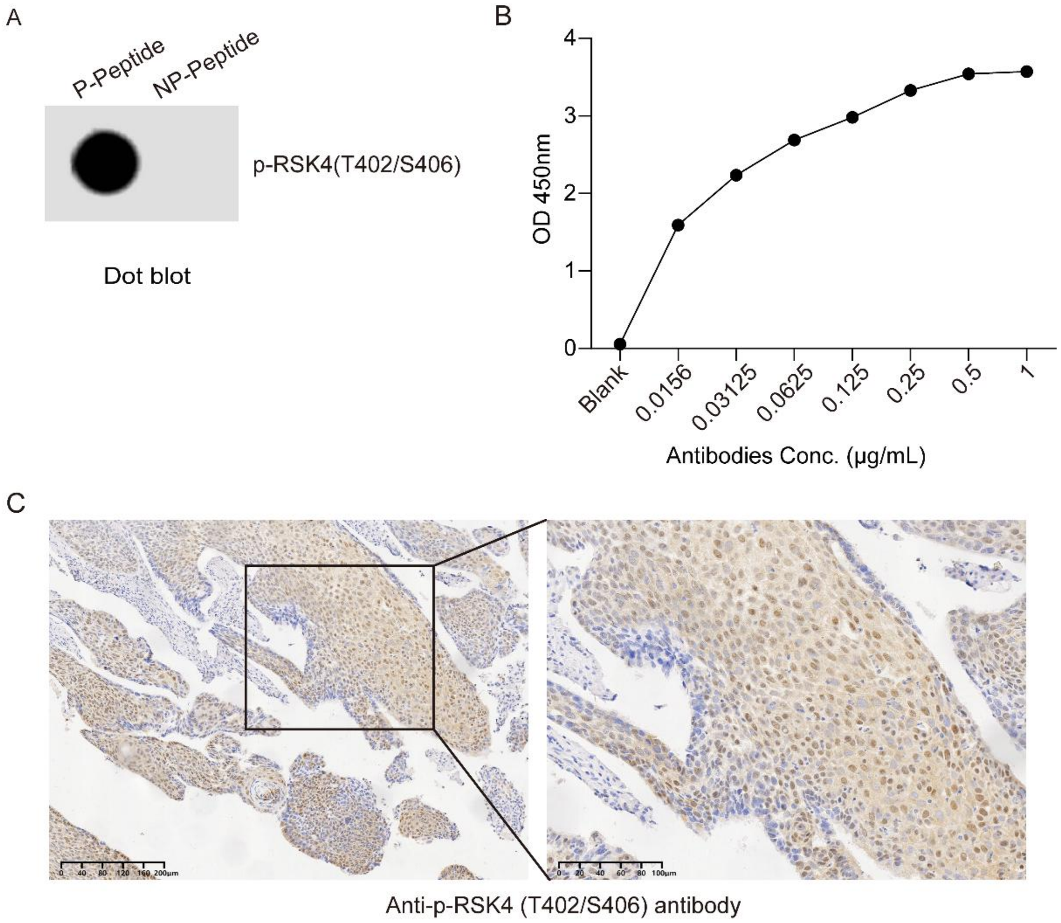

Supplementary Figure 2. Generation and identification of an p-RSK4 (T402/S406) antibody. (A) The specificity of the anti-p-RSK4 (T402/S406) antibody was verified by dot blot. 50ng of phospho-peptide or non-phospho-peptide per dot were adsorbed. Antibodies working concentration was 0.5μg/mL. **(**B) The specificity of the anti-p-RSK4 (T402/S406) antibody was confirmed using ELISA. **(**C) One section of ESCC tissues was incubated with anti-p-RSK4 (T402/S406) antibody to validate the specificity of anti-p-RSK4 (T402/S406) antibody for IHC staining.

Supplementary Table 1. Primer sequences used in the study

Primer name	Sequence (5'-3')
Primers for shRNA:	
shRSK4 #1	GATCCGGGTAAATGGTCTTAAAATGTCAAGAGCATTTTAAGACCATTTACCTTTTTTGGAATT
shRSK4 #2	GATCCGGCTCCTGAAGTAGTAAATATCAAGAGTATTTACTACTTCAGGAGCTTTTTTGGAATT
shGSK3β #1	GATCCGGCTCAAGGTGGTAGGCTACTTCCTGTCAGATAGCCTACCACCTTGAGCCTTTTTG
shGSK3β #2	GATCCCCAGGTAGATGATCATTTACTTCCTGTCAGATAAATGATCATCTACCTGGTTTTTG
shFBXW7 #1	CGGCCGGATCTCTTGATACATCAACTCGAGTTGATGTATCAAGAGATCCTTTTTG
shFBXW7 #2	CGGCCGGTAGAGGAGGAACAGCAACTCGAGTTGCTGTTCCTCCTCTACCTTTTTG
shOGT #1	CGGGCTGAGCAGTATTCCGAGAAACTCGAGTTTCTCGGAATACTGCTCAGCTTTTTG
shOGT #2	CGGGCCCTAAGTTTGAGTCCAAATCTCGAGATTTGGACTCAAACTTAGGGCTTTTTG
sgRNAs targeting	
sgRSK4 #1	TCAGCAGTATACCGCTGATG
sgRSK4 #2	GCGAGCAGCGGCGAGGTAAA

Supplementary Table 2. Antibody information used in immunohistochemistry and western blot

Protein	Application	Origin	Dilution
RSK4	IHC & WB	HPA003904, Sigma-Aldrich	1:50 & 1:200
p-GSK-3β (Ser9)	WB	#5558, Cell Signaling Technology	1:1000
FBXW7	WB	sc-293423, Santa Cruz Biotechnology	1:1000
OGT	WB	11576-2-AP, Proteintech Group	1:1000
OGA	WB	ab124807, Abcam	1:1000
O-linked N-acetylglucosamine (O-GlcNAc)	IHC & WB	#MA1-072, Invitrogen	:100 & 1:1000
Ki-67	IHC	MAB-0672, MXB Biotechnologies	1:200
SOX2	WB	#3579, Cell Signaling Technology	1:1000
CD271	WB	55014-1-AP, Proteintech Group	1:1000
ABCG2	WB	10051-1-AP, Proteintech Group	1:500
GSK-3β	WB	#12456, Cell Signaling Technology	1:1000
Cleaved Caspase-3	IHC	#9664, Cell Signaling Technology	1:500
Ubiquitin	WB	#20326, Cell Signaling Technology	1:1000
β-tubulin	WB	KM9003, Sungene Biotech	1:3000
HA-Tag	WB	#3724, Cell Signaling Technology	1:1000
Flag-Tag	WB	#8146, Cell Signaling Technology	1:1000
Flag-Tag	WB	#14793, Cell Signaling Technology	1:1000
Myc-Tag	WB	#13987, Cell Signaling Technology	1:1000
His-Tag	WB	#2366, Cell Signaling Technology	1:1000
V5-Tag	WB	#13202, Cell Signaling Technology	1:1000
RPS6	WB	#2217, Cell Signaling Technology	1:1000
p-RPS6	WB	#4858, Cell Signaling Technology	1:2000

Abbreviations: IHC, immunohistochemistry; WB, western blot.

Supplementary Table 4. YinOYang 1.2 predicted O-β-GlcNAc sites of RSK4

Residue	O-GlcNAc	Potential
405	T	0.5501
700	S	0.4734
729	S	0.4718
742	S	0.4296
730	S	0.4108
718	T	0.4049
378	S	0.3932
251	S	0.3871
368	T	0.3852
585	T	0.3841
743	T	0.382
710	T	0.3787
372	S	0.375
394	S	0.3709
402	T	0.3671
736	S	0.3573
555	S	0.3558
406	S	0.3526
521	S	0.3367
614	T	0.3289
741	T	0.3208
365	T	0.3196
302	S	0.3151
258	S	0.3146
547	S	0.3133
620	T	0.3126
528	S	0.3056
448	T	0.3019
560	S	0.2972
25	S	0.2957
661	S	0.2897
497	T	0.2873
653	S	0.2837
351	S	0.2821
94	T	0.2799
19	S	0.2753
58	T	0.2726
436	S	0.2706
447	T	0.2626
673	T	0.2599
712	S	0.2592

268	T	0.2559
24	S	0.2551
144	T	0.2508
393	T	0.2496
253	S	0.2486
681	S	0.2467
357	T	0.2454
482	T	0.239
389	S	0.2388
715	T	0.2367
557	S	0.235
171	T	0.2342
232	S	0.2332
607	S	0.233
120	T	0.2308
297	S	0.2307
83	S	0.2273
530	T	0.2268
223	S	0.2267
465	S	0.2239
438	S	0.2229
643	S	0.2229
581	T	0.222
236	T	0.2149
516	S	0.2101
111	S	0.2084
645	S	0.2056
629	T	0.2041
281	T	0.2039
162	T	0.2017
684	T	0.2017
220	S	0.2014
318	S	0.1989
165	S	0.1987
460	S	0.1912
45	S	0.1817
215	T	0.175
270	T	0.1733

Supplementary Table 5. Crosstabulated of O-GlcNAcylation immunostaining vs clinicalpathological features of ESCC cases in Xijing cohort

Features (n)	O-GlcNAcylation		*P* value
	Positive (n, %)	Negative (n, %)	
Gender			0.179
Male (123)	65 (52.8%)	58 (47.2%)	
Female (60)	38 (63.3%)	22 (56.7%)	
Age (years)			0.570
⩾60 (94)	51 (54.3%)	43 (45.7%)	
＜60 (89)	52 (58.4%)	37 (41.6%)	
Tumour size (cm)			0.595
＞3.0 (92)	50 (54.4%)	42 (45.6%)	
⩽3.0 (91)	53 (58.2%)	38 (41.8%)	
pTNM stage			0.237
Ⅰ-Ⅱ (87)	45 (51.7%)	42 (48.2%)	
Ⅲ-Ⅳ (96)	58 (60.4%)	38 (39.6%)	
Grade			0.934
1 (99)	56 (56.6%)	43 (43.4%)	
2-3 (84)	47 (56.0%)	37 (44.0%)	
T stage			0.229
1-2 (96)	50 (52.1%)	46 (47.9%)	
3-4 (87)	53 (60.9%)	34 (39.1%)	
N stage			**0.032**
0 (82)	39 (47.6%)	43 (52.4%)	
1-3 (101)	64 (63.4%)	37 (36.6%)	
Vascular invasion (CD31)			**0.005**
Yes (83)	56 (67.5%)	27 (32.5%)	
No (100)	47 (47.0%)	53 (53.0%)	
Nerve invasion (S-100)			0.845
Yes (90)	50 (55.6%)	40 (44.4%)	
No (93)	53 (57.0%)	40 (43.0%)	

NOTE. Bold values indicate statistical significance.

Abbreviations: ESCC, esophageal squamous cell carcinoma; pTNM, UICC pathological TNM classification for ESCC (8th edition); T, tumor; N, lymph node.

Supplementary Table 6. Multivariate Cox analysis of overall survival and progression-free survival of ESCC cases in Xijing cohort with follow-up information

Features	Overall Survival		Progression-free survival	
	HR (95% CI)	*P* value	HR (95% CI)	*P* value
Gender (male vs female)	0.916 (0.465-1.804)	0.799	1.110 (0.603-2.040)	0.738
Age (＞60 vs ≤60 years)	0.663 (0.353-1.246)	0.202	0.762 (0.426-1.364)	0.360
Tumour size (＞3 vs ≤3cm)	0.905 (0.486-1.687)	0.754	0.872 (0.489-1.556)	0.643
pTNM stage (Ⅲ/Ⅳ vs Ⅰ/Ⅱ)	1.311 (0.702-2.450)	0.395	1.178 (0.656-2.117)	0.583
Grade (2/3 vs 1)	1.154 (0.620-2.150)	0.651	1.250 (0.697-2.241)	0.454
T stage (3/4 vs 1/2)	2.392 (1.254-4.563)	**0.008**	1.894 (1.052-3.409)	**0.033**
N stage (≥1 vs 0）	1.113 (0.597-2.077)	0.736	1.068 (0.596-1.912)	0.825
Vascular invasion	1.429 (1.223-1.827)	**0.011**	1.420 (1.230-1.767)	**0.005**
Nerve invasion	1.566 (0.833-2.944)	0.164	1.467 (0.814-2.643)	0.202
O-GlcNAcylation (high vs low)	2.546 (1.090-5.948)	**0.031**	2.549 (1.134-5.729)	**0.024**
p-RSK4 (T402/S406) (high vs low)	0.639 (0.280-0.985)	**0.003**	0.590 (0.270-0.917)	**0.001**

NOTE. Bold values indicate statistical significance.

Abbreviations: ESCC, esophageal squamous cell carcinoma; HR, hazard ratio; 95% CI, 95% confidential interval; pTNM, UICC pathological TNM classification for ESCC (8th edition); T, tumor; N, lymph node.

Supplementary Table 7. Crosstabulated of p-RSK4 (T402/S406) immunostaining vs clinicalpathological features of ESCC cases in Xijing cohort

Features (n)	p-RSK4(T402/S406)		*P* value
	Positive (n, %)	Negative (n, %)	
Gender			0.877
Male (123)	63 (51.2%)	60 (48.8%)	
Female (60)	30 (50.0%)	30 (50.0%)	
Age (years)			0.211
⩾60 (94)	52 (55.3%)	42 (44.7%)	
＜60 (89)	41 (46.1%)	48 (53.9%)	
Tumour size (cm)			0.415
＞3.0 (92)	44 (47.8%)	48 (52.2%)	
⩽3.0 (91)	49 (53.8%)	42 (46.2%)	
pTNM stage			0.262
Ⅰ-Ⅱ (87)	48 (55.2%)	39 (44.8%)	
Ⅲ-Ⅳ (96)	45 46.9%)	51 (53.1%)	
Grade			0.115
1 (99)	45 (45.5%)	54 (54.5%)	
2-3 (84)	48 (57.1%)	36 (42.9%)	
T stage			0.341
1-2 (96)	52 (54.2%)	44 (45.8%)	
3-4 (87)	41 (47.1%)	46 (52.9%)	
N stage			**0.013**
0 (82)	50 (61.0%)	32 (39.0%)	
1-3 (101)	43 (42.6%)	58 (57.4%)	
Vascular invasion (CD31)			**0.015**
Yes (83)	34 (41.0%)	49 (59.0%)	
No (100)	59 (59.0%)	41 (41.0%)	
Nerve invasion (S-100)			0.827
Yes (90)	45 (50.0%)	45 (50.0%)	
No (93)	48 (51.6%)	45 (48.4%)	

NOTE. Bold values indicate statistical significance.

Abbreviations: ESCC, esophageal squamous cell carcinoma; pTNM, UICC pathological TNM classification for ESCC (8th edition); T, tumor; N, lymph node.
